# Supplementary material for: Genome-wide association study identifies five risk loci for pernicious anemia
Source: Nat Commun. 2021 Jun 18;12:3761. doi: 10.1038/s41467-021-24051-6 (PMC8213695; doi:10.1038/s41467-021-24051-6)
Supplement: Supplementary file 1 — Supplementary Information [file 41467_2021_24051_MOESM1_ESM.pdf]

Supplementary Information

Genome-wide association study identifies five risk loci for pernicious anemia

Laisk et al.

Supplementary Table 1. Association statistics for lead variants in each locus in cohort level analysis

		Meta-analysis_BOTH						Males			Females			GWAMA gender heterogeneity p-value
Variant	Cohort	EA	NEA	PVAL	BETA	SE	INFO	PVAL	BETA	SE	PVAL	BETA	SE	
rs6679677	EstBB	A	C	1.26E-04	0.41	0.11	1	5.29E-01	0.14	0.23	5.07E-05	0.49	0.12	
	UKBB	A	C	2.70E-08	0.49	0.09	1	8.37E-05	0.47	0.12	4.83E-04	0.29	0.08	
	FinnGen	A	C	1.02E-14	0.51	0.07	>0.6	NA	NA	NA	NA	NA	NA	
	Meta-analysis	A	C	1.91E-24	0.49	0.05		1.64E-04	0.40	0.11	2.43E-07	0.35	0.07	6.99E-01
rs12616502	EstBB	A	G	5.54E-04	0.56	0.16	0.99	6.15E-04	1.27	0.37	3.11E-02	0.39	0.18	
	UKBB	A	G	1.70E-05	0.52	0.12	0.99	5.35E-05	0.69	0.17	7.42E-03	0.30	0.11	
	FinnGen	NA	NA	NA	NA	NA	NA	NA	NA	NA	NA	NA	NA	
	Meta-analysis	A	G	3.14E-08	0.53	0.10		3.38E-07	0.80	0.16	6.41E-04	0.33	0.10	1.06E-02
rs28414666	EstBB	G	A	1.78E-03	0.27	0.09	0.99	8.42E-03	0.50	0.19	2.83E-02	0.21	0.10	
	UKBB	G	A	4.40E-07	0.33	0.07	0.99	1.53E-05	0.39	0.09	1.16E-05	0.27	0.06	
	FinnGen	G	A	8.00E-09	0.33	0.06	>0.6	NA	NA	NA	NA	NA	NA	
	Meta-analysis	G	A	1.40E-16	0.32	0.04		4.81E-07	0.41	0.08	1.08E-06	0.25	0.05	1.10E-01
rs2476491	EstBB	A	T	7.17E-05	0.32	0.08	0.99	2.09E-02	0.39	0.17	6.68E-04	0.30	0.09	
	UKBB	A	T	7.80E-04	0.19	0.06	0.99	1.13E-01	0.13	0.08	3.17E-03	0.16	0.05	
	FinnGen	A	T	4.97E-03	0.15	0.05	>0.6	NA	NA	NA	NA	NA	NA	
	Meta-analysis	A	T	1.90E-08	0.20	0.03		1.60E-02	0.17	0.07	1.75E-05	0.20	0.05	7.45E-01
rs74203920	EstBB	T	C	1.85E-05	1.32	0.31	1	2.77E-02	1.37	0.62	4.68E-05	1.43	0.35	
	UKBB	T	C	4.00E-02	0.45	0.22	0.96	9.91E-02	0.49	0.30	1.73E-01	0.29	0.21	
	FinnGen	T	C	1.08E-05	0.54	0.12	>0.6	NA	NA	NA	NA	NA	NA	
	Meta-analysis	T	C	2.33E-09	0.60	0.10		1.46E-02	0.66	0.27	1.11E-03	0.59	0.18	8.36E-01

The FinnGen summary statistics file only includes variants with an imputation INFO score >0.6. Individual level data is not available for FinnGen data, therefore sex-stratified analyses for lead signals were restricted to EstBB and UKBB data.

Supplementary Figures

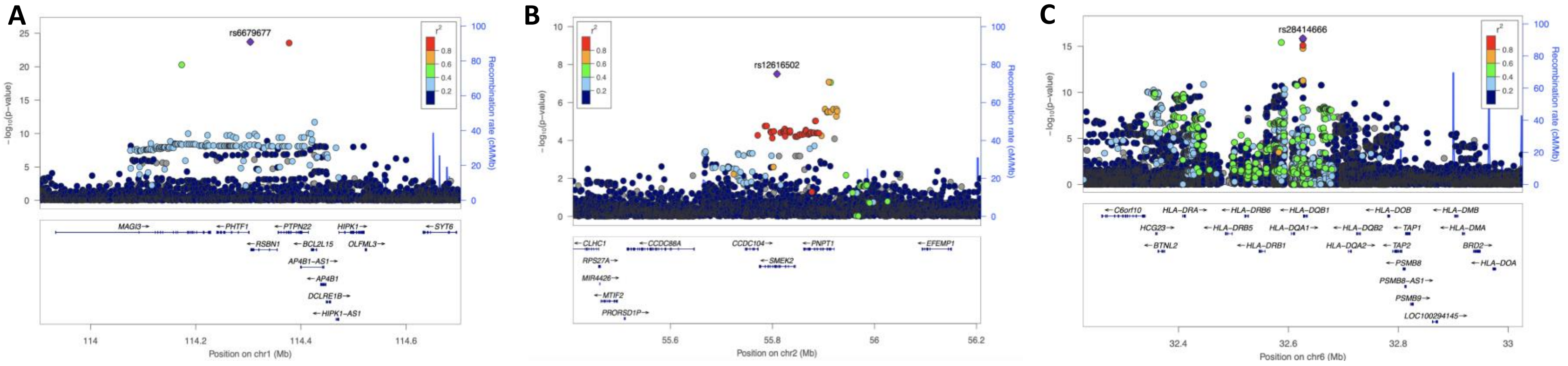

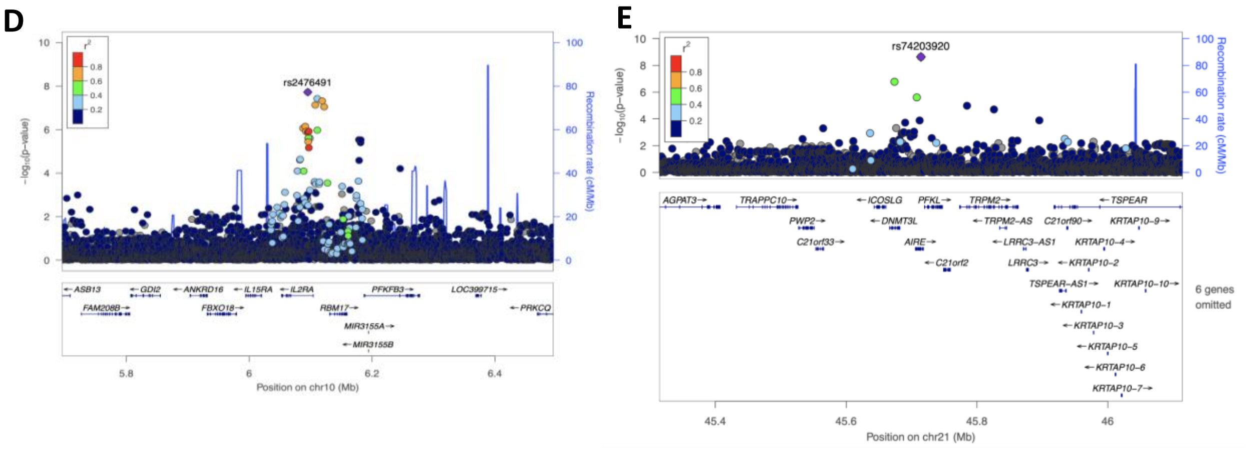

Supplementary Figure 1. Regional plots of five association signals from pernicious anemia GWAS meta-analysis. A) rs6679677 on chr1; B) rs12616502 on chr2; C) rs28414666 on chr6; D) rs2476491 on chr10; and E) rs74203920 on chr21

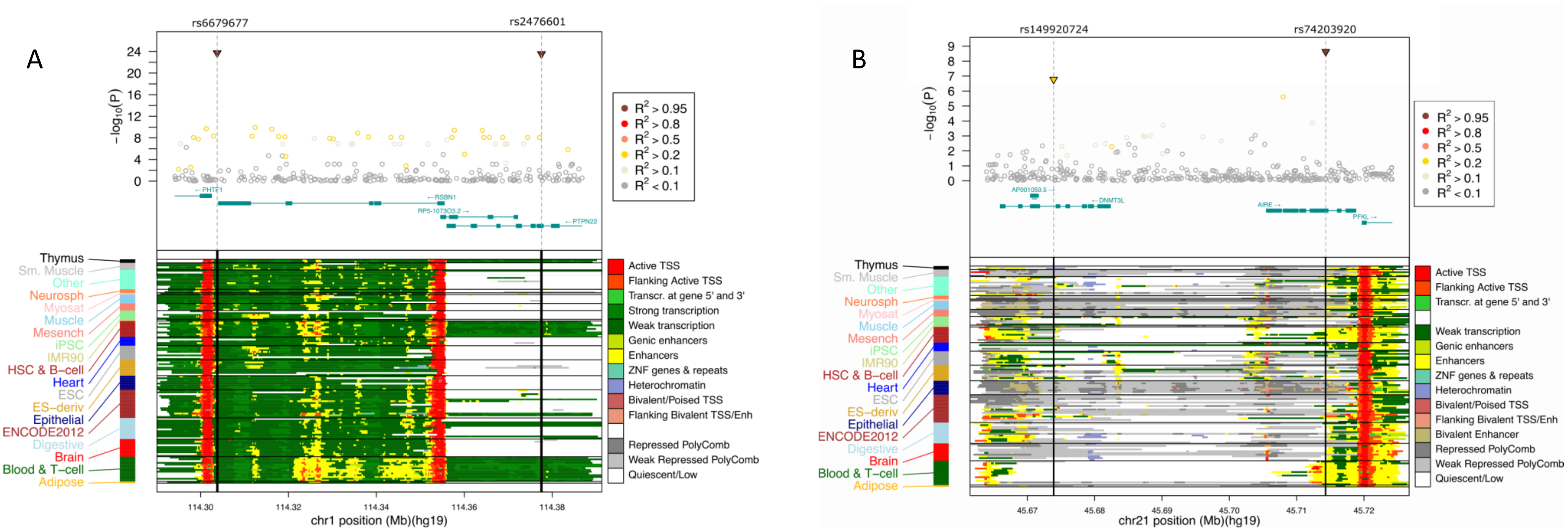

Supplementary Figure 2. Functional annotation of the credible set variants on chromosomes 1 (A) and 21 (B) On the right, the top panel depicts regional association statistics from meta-analysis. Credible set variants are marked with triangles and the color coding of each variant corresponds to the LD pattern in the region (see legend in the figure). The bottom panels show 15-state chromatin marks for 127 samples from Roadmap Epigenomics project (see legend in the figure for color coding).

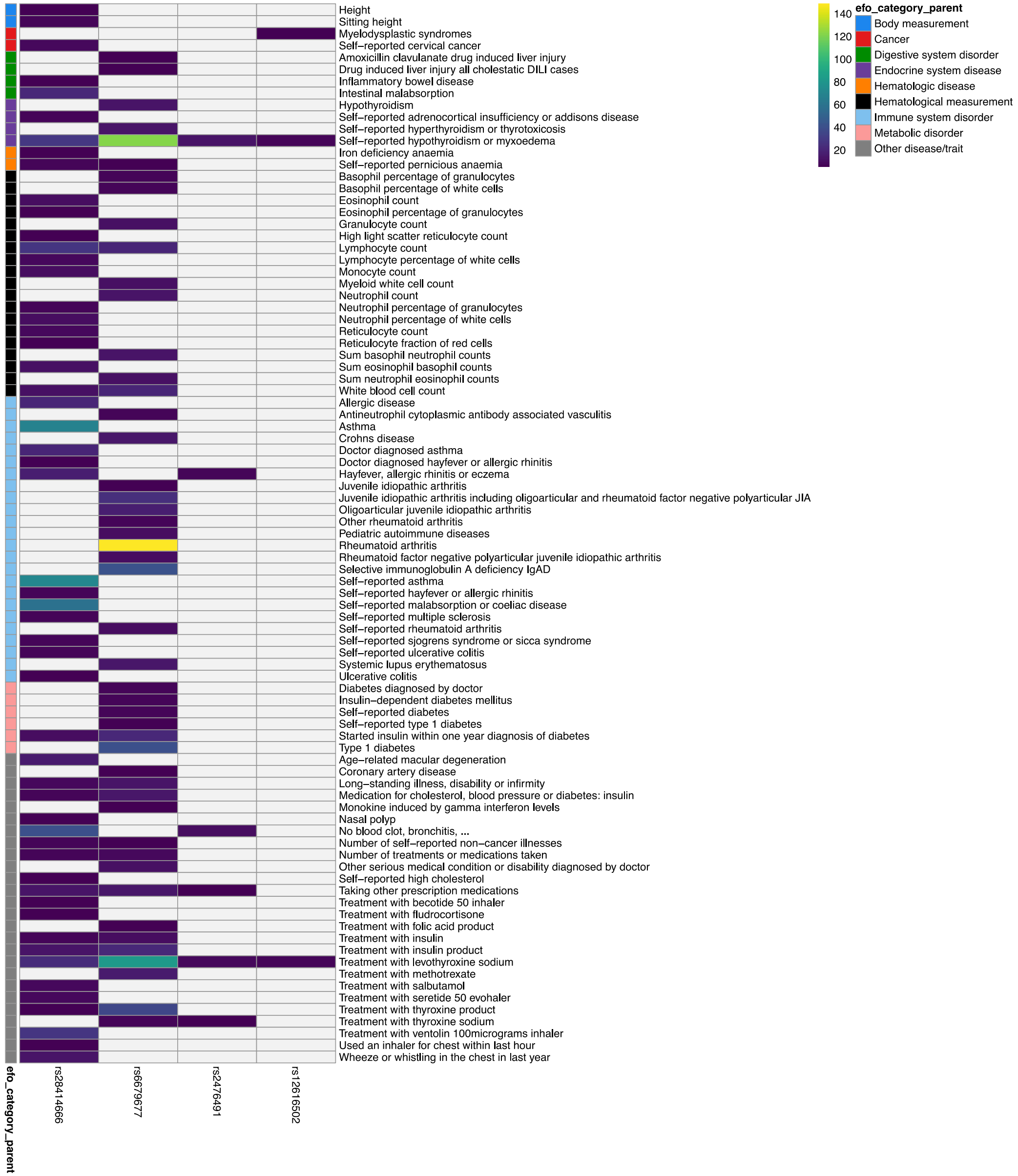

Supplementary Figure 3. Heatmap of the Phenoscanner look-up. Lead signals of each locus (that were present in Phenoscanner) are shown in columns, traits in rows, which have been grouped by EFO parent categories (see color legend). Cells are coloured by strength of association (see legend)

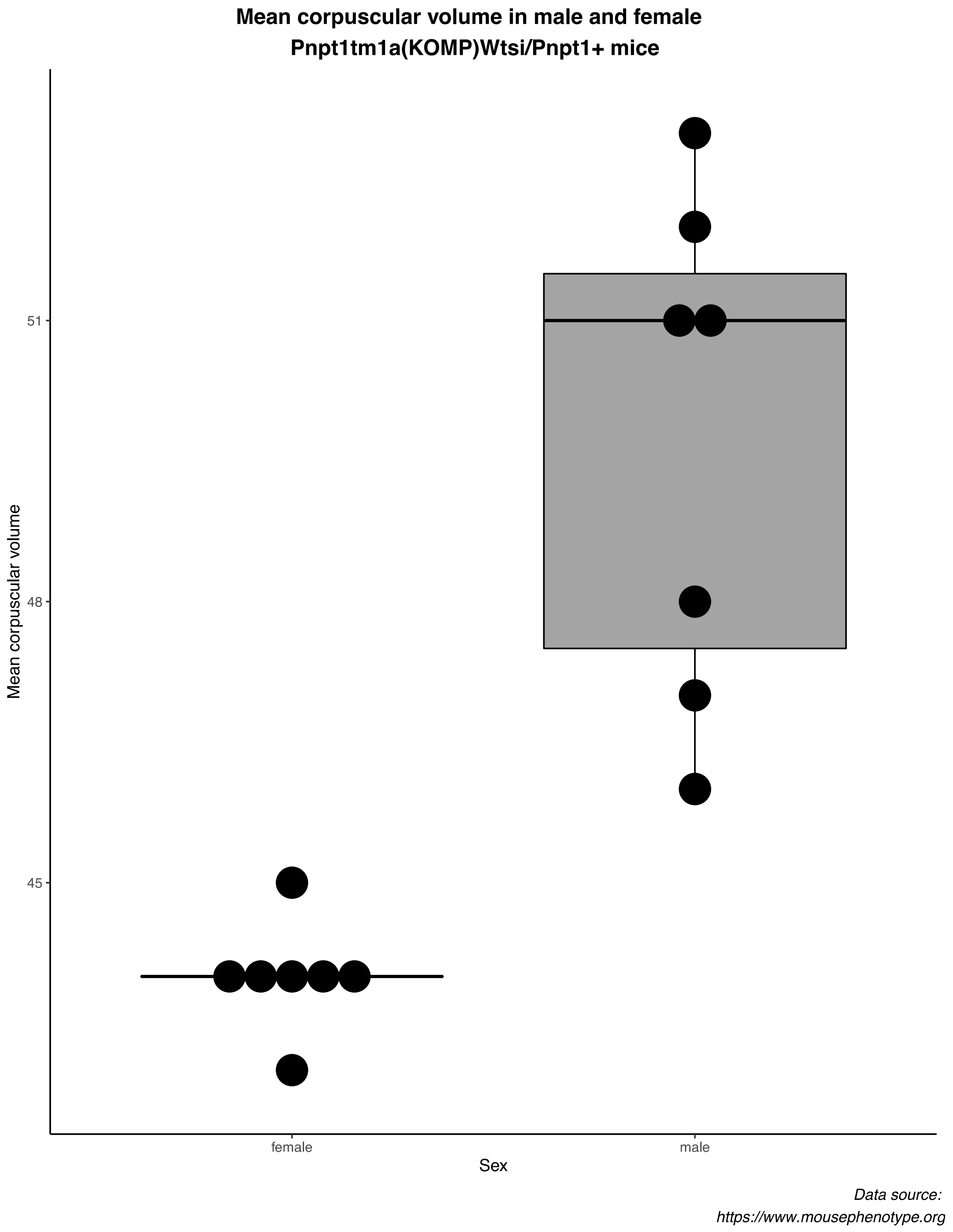

Supplementary Figure 4. Mean corpuscular volume in male and female Pnpt1tm1a(KOMP)Wtsi/Pnpt1+ mice.
