# Supplementary material for: Genome-wide association study identifies five risk loci for pernicious anemia
Source: Nat Commun. 2021 Jun 18;12:3761. doi: 10.1038/s41467-021-24051-6 (PMC8213695; doi:10.1038/s41467-021-24051-6)
Supplement: Supplementary file 3 — Description of Additional Supplementary Files [file 41467_2021_24051_MOESM3_ESM.pdf]

Description of Additional Supplementary Files

File Name: Supplementary Data 1
Description: Results of the coloc analysis (.xlsx)

File Name: Supplementary Data 2
Description: Results of the look-up in GWAS catalogue (.xlsx)

File Name: Supplementary Data 3
Description: Results of the phenoscanner look-up (.xlsx)

File Name: Supplementary Data 4
Description: Data on mean corpuscular volume from mouse PNPT1 mutants from International Mouse Phenotyping Consortium database (.xlsx)

File Name: Supplementary Data 5
Description: Association statistics for variants associated with relevant associated phenotypes (.xlsx)

File Name: Supplementary Data 6
Description: Diagnosis codes associated with pernicious anemia (.xlsx)

File Name: Supplementary Data 7
Description: List of autoimmune disease codes (.xlsx)
