# Supplementary material for: Genome-wide association study identifies five risk loci for pernicious anemia
Source: Nat Commun. 2021 Jun 18;12:3761. doi: 10.1038/s41467-021-24051-6 (PMC8213695; doi:10.1038/s41467-021-24051-6)
Supplement: Supplementary file 11 — Reporting Summary [file 41467_2021_24051_MOESM11_ESM.pdf]

nature research

Corresponding author(s): T. Laisk

Last updated by author(s): Apr 23, 2021

Reporting Summary

Nature Research wishes to improve the reproducibility of the work that we publish. This form provides structure for consistency and transparency in reporting. For further information on Nature Research policies, see our Editorial Policies and the Editorial Policy Checklist.

Statistics

For all statistical analyses, confirm that the following items are present in the figure legend, table legend, main text, or Methods section.

n/a	Confirmed	
☐	☒	The exact sample size (*n*) for each experimental group/condition, given as a discrete number and unit of measurement
☒	☐	A statement on whether measurements were taken from distinct samples or whether the same sample was measured repeatedly
☐	☒	The statistical test(s) used AND whether they are one- or two-sided<br>*Only common tests should be described solely by name; describe more complex techniques in the Methods section.*
☐	☒	A description of all covariates tested
☐	☒	A description of any assumptions or corrections, such as tests of normality and adjustment for multiple comparisons
☐	☒	A full description of the statistical parameters including central tendency (e.g. means) or other basic estimates (e.g. regression coefficient) AND variation (e.g. standard deviation) or associated estimates of uncertainty (e.g. confidence intervals)
☐	☒	For null hypothesis testing, the test statistic (e.g. *F*, *t*, *r*) with confidence intervals, effect sizes, degrees of freedom and *P* value noted<br>*Give P values as exact values whenever suitable.*
☐	☒	For Bayesian analysis, information on the choice of priors and Markov chain Monte Carlo settings
☒	☐	For hierarchical and complex designs, identification of the appropriate level for tests and full reporting of outcomes
☐	☒	Estimates of effect sizes (e.g. Cohen's *d*, Pearson's *r*), indicating how they were calculated

Our web collection on statistics for biologists contains articles on many of the points above.

Software and code

Policy information about availability of computer code

Data collection	Information on how genotype and phenotype data was collected for each cohort is available in Methods and for FinnGen and UKBB from the respective PheWeb pages. No specific software was used for data collection.
Data analysis	In Estonian Biobank, GenomeStudio (v2.0.4), Eagle (v2.3) and Beagle (v28Sep18.793) were used as part of the standard genotyping and imputation pipeline. Cohort-level analyses were carried out with SAIGE (v0.38). HLA allele imputation was carried out with SNP2HLA 1.0.3. Central meta-analysis was conducted using the GWAMA software (v2.2.2). Finemapping was carried out with R package corrcoverage v1.2.1 (https://annahutch.github.io/corrcoverage/index.html). Inkscape 1.1.0-dev (0486c1a, 2020-10-10) was used for finetuning the figures. For colocalisation, COLOC (v.3.2.1) was used and results were visualised with LocusCompareR (v1.0.0) library. FUMA v1.3.6a was used for GWAS catalogue (e91_r2018-02-06) look-up. PhenoScanner v2 was used for look-up of phenotype associations for the GWAS lead variants in previous GWAS studies, using the phenoscanner (v1.0) R package, and the results were visualised using pheatmap library in R 3.6.1. and a modified script from (https://github.com/LappalainenLab/spiromics-covid19-eqtl/blob/master/eqtl/summary_phenoscanner_lookup.Rmd. Associated phenotypes analysis was visualised with the PheWas library (0.99.5-4) (https://github.com/PheWAS/PheWAS). All other analyses were conducted in R 3.6.1.

For manuscripts utilizing custom algorithms or software that are central to the research but not yet described in published literature, software must be made available to editors and reviewers. We strongly encourage code deposition in a community repository (e.g. GitHub). See the Nature Research guidelines for submitting code & software for further information.

Data

Policy information about availability of data

All manuscripts must include a data availability statement. This statement should provide the following information, where applicable:

- Accession codes, unique identifiers, or web links for publicly available datasets
- A list of figures that have associated raw data
- A description of any restrictions on data availability

Used UKBB and FinnGen summary statistics can be browsed and downloaded from UKBB PheWeb (http://pheweb.sph.umich.edu/SAIGE-UKB/) and FinnGen PheWeb (http://r3.finngen.fi), respectively. Full meta-analysis summary statistics can be downloaded from http://www.geenivaramu.ee/tools/pernicious_anemia_Laisketal2021_sumstats.gz. All GWAS analyses and meta-analysis were carried out with standard tools and pipelines. The analyses in this paper also use data from the Mouse Genome Database: http://www.informatics.jax.org; International Mouse Phenotyping Consortium: https://www.mousephenotype.org; GTEx Portal: https://gtexportal.org/home/; eQTL Catalogue: https://www.ebi.ac.uk/eqtl/; GWAS Catalog: https://www.ebi.ac.uk/gwas/; Roadmap Epigenomics project (http://egg2.wustl.edu/roadmap/data/byFileType/chromhmmSegmentations/ChmmModels/coreMarks/jointModel/final/)

Field-specific reporting

Please select the one below that is the best fit for your research. If you are not sure, read the appropriate sections before making your selection.

- [x] Life sciences
- [ ] Behavioural & social sciences
- [ ] Ecological, evolutionary & environmental sciences

For a reference copy of the document with all sections, see nature.com/documents/nr-reporting-summary-flat.pdf

Life sciences study design

All studies must disclose on these points even when the disclosure is negative.

Sample size	We used all publicly available summary statistics (UKBB and FinnGen) for this phenotype and individual level from Estonian Biobank. Our analysis included 2,166 cases and 659,516 European controls from population-based biobanks. Pernicious anemia is a rare condition and by including all available data from population-based biobanks, we have achieved the largest sample size currently possible.
Data exclusions	Details on QC filters used in EstBB data can be found in the Methods. According to the EstBB genotyping and imputation pipeline, individuals were excluded from the analysis if their call-rate was < 95% or if sex defined based on heterozygosity of X chromosome did not match sex in phenotype data. Before imputation, variants were filtered by call-rate < 95%, HWE p-value < 1e-4 (autosomal variants only), and minor allele frequency < 1%. These criteria were applied to ensure quality of the resulting genotype data. All exclusion criteria were pre-established. For FinnGen and UKBB we used publicly GWAS summary statistics and applied no additional exclusion filters for these datasets. In the follow-up analysis, we used UKBB data. In these analyses we focused on samples of genetically confirmed British European ancestry. We excluded individuals who had withdrawn their consent, were labelled with poor heterozygosity or missingness as defined by UKBB, had excess (>10) relatives, were not included in autosome phasing, had putative sex chromosome aneuploidy, or had sex mismatch between self-reported and genotype data. Pernicious anemia cases were extracted using the ICD10 D51.0 code in HES (Hospital Episodes and Spells) data (downloaded on July 11th, 2020). All exclusion criteria were pre-established.
Replication	Replication was not attempted as there were no sufficiently sized cohorts available for replication because pernicious anemia is a rare condition. However, the main findings were consistent (in terms of effect estimates) across cohorts.
Randomization	The study is a case-control GWAS study/meta-analysis, therefore subjects were not randomly allocated to study groups. The following covariates were included in genetic association analyses: EstBB - sex, year of birth, 10 genetic principical components; UKBB - sex, year of birth, 4 genetic PCs; FinnGen - sex, age, 10 PCs, genotyping batch
Blinding	The study is a case-control GWAS study/meta-analysis, therefore blinding is not relevant to this study, as disease status (presence of specific ICD code) is the basis for forming the groups. GWAS meta-analysis was conducted using summary statistics with no individual-level data.

Reporting for specific materials, systems and methods

We require information from authors about some types of materials, experimental systems and methods used in many studies. Here, indicate whether each material, system or method listed is relevant to your study. If you are not sure if a list item applies to your research, read the appropriate section before selecting a response.

Materials & experimental systems

n/a	Involved in the study
☒	☐ Antibodies
☒	☐ Eukaryotic cell lines
☒	☐ Palaeontology and archaeology
☒	☐ Animals and other organisms
☐	☒ Human research participants
☒	☐ Clinical data
☒	☐ Dual use research of concern

Methods

n/a	Involved in the study
☒	☐ ChIP-seq
☒	☐ Flow cytometry
☒	☐ MRI-based neuroimaging

Human research participants

Policy information about studies involving human research participants

Population characteristics

Our analysis 2,166 cases with pernicious anemia and 659,516 European controls from population-based biobanks. In EstBB, individuals with pernicious anemia were identified using the ICD10 code D51.0 and all biobank participants who did not have this diagnosis were considered as controls. Individuals with pernicious anemia were identified using the ICD-10 code D51.0, resulting in 378 cases (22% males (age at baseline 63.5± 15.7 years) and 78% females (54.9 ± 15.9 years)) and 138,207 controls (34% males (43.1±16.2 years) and 66 % females (44.0±16.0 years)) for analysis. Information on ICD codes is obtained via regular linking with the national Health Insurance Fund and other relevant national databases. The UK Biobank (UKBB) is a prospective cohort of 502,637 individuals aged 37-73 recruited in 2006-2010 from across the UK, who completed detailed questionnaires regarding socio-demographic and lifestyle characteristics and their medical history, and had a clinical assessment. Additional information about medical conditions (both existing at baseline and occurring during follow-up) has been obtained through linking with hospital admission and mortality data. Full details of the study have been reported in [PMID: 25826379]. Publicly available GWAS summary statistics downloaded from the UKBB PheWeb [http://pheweb.sph.umich.edu/SAIGE-UKB/about] were used for the analysis. Briefly, the PheWeb includes GWAS summary statistics for ICD code-based traits extracted from electronic health records. Phenotypes have been classified into 1,403 broad PheWAS codes, including pernicious anemia (PheCode 281.11), defined using the ICD-10 code D51.0 and excluding other anemias under the PheCodes 280-285.99. For additional analyses requiring individual-level data (cohort descriptive statistics, sex-stratified analysis of lead signals and look-up of additional autoimmune diseases in pernicious anemia cases, we used data under the application 17085. Since the phenotype data was extracted at different timepoints and using slightly different (exclusion) criteria, the follow-up analyses included a larger number of pernicious anemia cases (n=1,192) compared to the original GWAS analysis (n=754). The descriptive characteristics of the follow-up dataset in the UKBB were: cases – 384 men (age 61.9±15.7 years) and 808 women (age 59.1±7.7 years), controls 187,056 men (age 57.1±8.1 years) and 219,756 women (age 56.7±7.9 years).
FinnGen is a public-private partnership project combining data from Finnish biobanks and electronic health records from different registries. After a one-year embargo, the FinnGen summary stats are available for download. In this study, we used the results from the FinnGen release R3, which includes data from 135,638 individuals and more than 1,800 disease endpoints. The FinnGen disease endpoint "Vitamin B12 deficiency anemia" included all individuals with the ICD10 D51 diagnosis as cases. As we do not have access to individual level data of FinnGen participants, we do not know the age and gender distribution in this dataset.

Recruitment

The analyses were carried out using already existing data from voluntary unbiased (population-based) biobanks. Individuals corresponding to the criteria specified above were considered eligible for the study and no participants were specifically recruited for this study. In our analysis, cases were identified from population-based biobank data using the ICD-10 code for pernicious anemia (D51.0 or D51 in the FinnGen data). This approach was selected to simplify data analysis, but we acknowledge this approach may have some shortcomings. First, the used summary statistics for FinnGen cohort are for a broader vitamin B12 deficiency phenotype definition compared to other two cohorts (so it likely includes other cases of B12 deficiency). Second, the use of the code may vary in different healthcare systems, increasing heterogeneity of the phenotype. The potential misclassification of our control subjects as not having pernicious anemia can increase the heterogeneity in the analysed data and attenuate the results towards the null, meaning that either larger datasets with the current phenotype definition or further refinement of the phenotype definition is needed to increase the number of identified loci. At the same time, the prevalence of pernicious anemia in our studied datasets ranged from 0.2-0.8%, which is roughly in line with the expected prevalence of pernicious anemia (0.1% in the general population and >2% in over 60-year-olds)2.

Ethics oversight

In the EstBB, individual level data were analysed under approval 1.1-12/624 from the Estonian Committee on Bioethics and Human Research (Estonian Ministry of Social Affairs) and data release N05 from the EstBB. For UKBB and FinnGen, we used publicly available summary level data for genetic association analyses, so no separate ethics approval is necessary. For UKBB follow-up analyses, we used data under the application 17085 approved by the UKBB. No additional ethics approval were needed for this dataset and UKBB's Ethics and Governance Council provides guidelines for conducting studies with this dataset.

Note that full information on the approval of the study protocol must also be provided in the manuscript.
